# Ergonomic Improvements to Agricultural Harvest Baskets to Reduce the Risk of Musculoskeletal Disorders among Farmers

**DOI:** 10.3390/ijerph191710669

**Published:** 2022-08-26

**Authors:** Mintae Seo, Hyocher Kim, Wongeon Jung

**Affiliations:** National Institute of Agricultural Sciences, Rural Development Administration, 310 Nongsaengmyeong-ro, Deokjin-gu, Jeonju-si 54875, Korea

**Keywords:** musculoskeletal burden, ergonomics, electromyography, harvesting basket, farmer

## Abstract

Typical harvesting baskets (TB) are used in various agricultural workplaces; however, no study to date has reported their effect on the musculoskeletal system. Therefore, this study aimed to evaluate the effects of a novel basket with attached rotational handles (RHB) to help alleviate the work-related physical burden of farmers. We analyzed the surface electromyograms (EMGs) of seven muscles, evaluated the subjective discomfort levels and locally perceived discomfort (LPD) scores to investigate the discomfort in the whole body and seven hand muscles, respectively. The EMGs showed that muscle activity decreased in five muscles (flexor carpi ulnaris, extensor carpi radialis, lateral triceps, middle deltoid, and upper trapezius) and increased in two (biceps brachii and erector spinae) when the RHB was used (*p* < 0.05). The subjective discomfort score for the RHB decreased compared to that for TB (*p* < 0.001). The LPD scores also decreased, and the RHB and TB scores ranged from 1.25–1.40 and 3.1–3.25, respectively. The use of the RHB may prevent wrist bending, and reduce the activity of certain muscles while increasing the activity of other muscles. Therefore, it is necessary to conduct training and to evaluate the working posture while considering the affected muscles.

## 1. Introduction

A musculoskeletal disorder is a representative ergonomic problem for most industries. It has been reported that this type of disorder may be caused by workplace factors such as the use of excessive force, repetitive movements, improper work posture, the handling of heavy loads, contact with sharp surfaces, extreme high-/low-temperature environments, and whole-body/hand-arm vibrations [1,2,3]. Concerns regarding musculoskeletal disorders are increasing, particularly in industries in the manufacturing and construction sectors, as well as in industries where employees engage in relatively static tasks that feature working for long hours in a fixed posture, such as in a call center and a general office [4,5].

According to a survey by the Rural Development Administration in Korea in 2019, of a total of 91,694 farmers, the number of farmers with musculoskeletal disorders who took a day or more off from work was 77,613 (84.6%) [6]. There is a high possibility that the musculoskeletal burden in agriculture-related work is a result of factors such as an improper work posture or the sudden use of excessive force; furthermore, this burden could also be due mainly due to unpredictable or unstable work methods [7]. The risk of a musculoskeletal burden among farmers may also increase due to the use of tools or equipment that are unsuitable for the conditions or the task at hand. To reduce this burden, various studies are being conducted to improve or develop more convenient tools and equipment [8,9,10,11].

Typical harvesting baskets (TBs) are used for various purposes in addition to harvesting, such as to transport agricultural produce, materials, and tools throughout the farming workplace. While an awkward posture has been reported to be a cause of musculoskeletal disorders, only heavy weight or repetitive work was studied [7]. The handle of TBs is located on the upper left and right sides and is difficult to grasp, resulting in wrist bending among workers. TBs that are used primarily in Korean agricultural workplaces generally have a capacity of approximately 50 L, and can be filled with a weight of approximately 30 kg or more. If the weight is added, the load on the bent wrist will increase and the discomfort of a bent wrist may cause an improper posture. The pressure on upper body to support a heavy object may increase the musculoskeletal burden.

Musculoskeletal disorders in agricultural workplaces using these harvesting baskets have been studied in Korea. According to a previous study that targeted local viticulture farmers, the musculoskeletal burden assessment tool score (a rapid entire body assessment score) was higher during the movement of harvest baskets than during other tasks [12]. In another domestic study involving red pepper workers, the work-related burden caused by excessive force was investigated by analyzing detailed work factors, and the greatest labor burden was reported when lifting or moving objects of over 5 kg [13]. In addition, a previous study on local farmers working with vegetables cultivated in facilities reported that arthritis and disc herniation accounted for a larger proportion than other diseases such as hypertension [14]. Therefore, to reduce the risk of musculoskeletal disorders, replacement of a new handle, development of convenient equipment, or improvement in ergonomic management is needed.

Since the transport of heavy objects can also be considered a factor that contributes to these disorders, the development of tools and equipment that can help with transport may reduce the incidence of these disorders. Therefore, we performed quantitative electromyography (EMG) in this study, aiming to evaluate and compare the muscle activities of each body part of the agricultural site workers while using TBs, and while using an improved harvesting basket with rotational handles (RHB). In addition, the subjective discomfort that followed the action was analyzed to compare the effects of using each basket type. The quantitative evaluation results of the EMG and the data on the subjective discomfort levels reported with the rotational improvement in the handles of a TB may serve as a basis for the development of convenient equipment and the improvement in inappropriate working postures that may occur when handling heavy objects, thereby preventing musculoskeletal disorders in farm workers.

## 2. Materials and Methods

### 2.1. Participants

Four participants were included in the study: two women (ID: F1, F2) and two men (ID: M1, M2). These participants were not farmers, and had no history of musculoskeletal disorders. For EMG evaluation while using the harvesting basket, we measured the lengths of the arms, upper arm, and forearm (lower arm); the circumferences of the upper arm and wrist; and the length, width, and thickness of the hands of the participants. The standard definitions for measurements of body parts were used, as reported previously [15,16]. The arm length was defined as the distance from the lateral shoulder to the ulnar styloid through the radiale, the upper arm length was the distance from the lateral shoulder to the radiale, and the forearm length was the distance from the back olecranon to the ulnar styloid. The circumference of the upper arm was measured as the surface of the biceps brachii muscle, and the wrist circumference was the circumference of the radial styloid. The hand length was measured as the distance from the wrist line to the dactylion III. The measurements of the four participants are presented in Table 1.

### 2.2. Procedure

#### 2.2.1. Experimental Design

This was a quantitative evaluation using the EMG and an analysis of the subjective discomfort. Two types of baskets were evaluated: a TB used commonly in agricultural worksites and an RHB.

**Figure 1:**
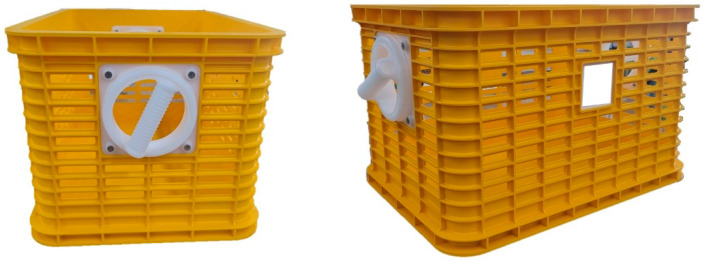
Harvesting baskets with rotational handles (RHB).

For the RHB used in the experiment, a prototype was manufactured to match the same level of actual work and weight as the harvest baskets using acrylonitrile butadiene styrene, which is a durable material. The handles were manufactured using poly-oxy-methylene, a material with a low friction coefficient, to minimize the friction caused by the rotation (Figure 1).

**Figure 2:**
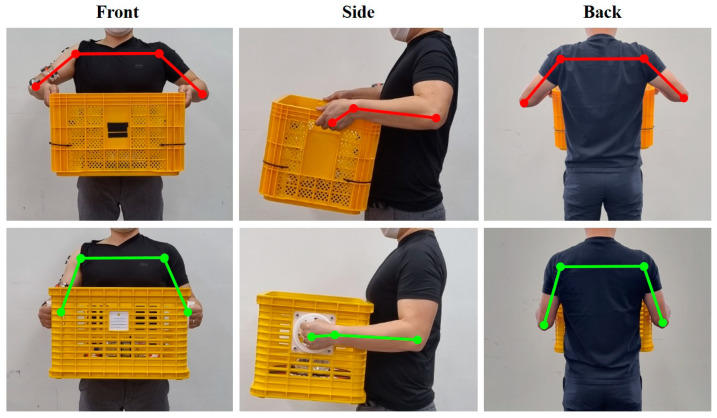
Basket types using in this experiment (red line, Typical Basket; green line, Rotational Handle Basket).

Both the basket types weighed 15 kg each. The four participants lifted the basket placed on the floor, moved it 5 m in a straight line, and then set it down on the floor. Each participant repeated the task five times for each basket type and responded immediately on the subjective discomfort level experienced after transporting the basket. After a task was performed for approximately 1 min, the participant had at least 5-min break. The changes in the body angle when using the TB and the RHB are shown in Figure 2.

#### 2.2.2. Electromyographic Measurement

**Figure 3:**
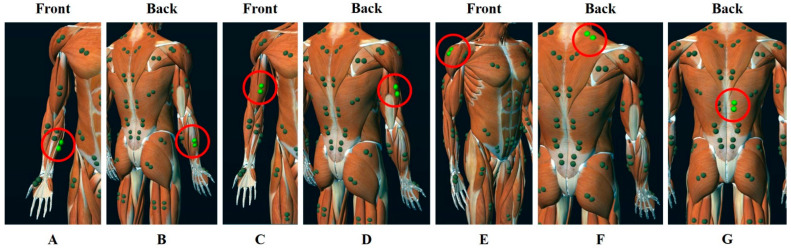
Location of electrodes (source: Noraxon MR3 3.12.42 software program, Noraxon Inc., Scottsdale, AZ, USA); (**A**) = Flexor Carpi Ulnaris, (**B**) = Extensor Carpi Radialis, (**C**) = Biceps Brachii, (**D**) = Lateral Triceps, (**E**) = Middle Deltoid, (**F**) = Upper Trapezius, (**G**) = Erector Spinae.

The muscle activity of each body region was measured using the EMG (Telemyo Desktop DTS System, Noraxon Inc., Scottsdale, AZ, USA; gain = 500, noise < 1 µV, sampling rate = 1500 Hz). The raw EMG data were processed using Noraxon MR3 3.12.42. software. The EMG signal was processed according to the following steps: filtering, rectification, and smoothing algorithm. The type of signal was band-pass, and the range of frequency was 20–400 Hz. Used filter was finite impulse response, and window were 79 points and lancosh. The smoothing algorithm was the root-mean-square and the window was 100 ms. Before the measurement were taken and after cleaning the attachment site with an alcohol swab, all the four participants had electrodes placed on them, parallel to the muscle fiber direction. An EMG evaluation was performed at seven different locations by attaching electrodes to the flexor carpi ulnaris, extensor carpi radialis, biceps brachii, lateral triceps, middle deltoid, upper trapezius, and erector spinae (Figure 3), with the attachment locations selected according to the SENIAM guidelines [15].

During the evaluation, the maximum voluntary contraction (MVC) was measured at the seven sites to standardize the different muscle activities of the participants, followed by the measurement of the muscle activity at each site according to the basket type. The MVC measurement for each muscle was measured as follows:

- Flexor carpi ulnaris: The participant’s hands were placed on the table with the palms facing down. When the technician pushed down the participant’s hands with their fists, the participant pushed them up simultaneously in the opposite direction (toward their own body).
- Extensor carpi radialis: The back of the participant’s hands was placed on the table facing down, then they were lifted high while maintaining clenched fists.
- Biceps brachii: The participant’s elbows were placed on the table, with their palm upward, and when the technician pulled the elbows toward them, the participant pulled in the opposite direction toward themselves.
- Lateral triceps: Same as above, but the technician and study participant pushed against each other.
- Middle deltoid: After the study participant was seated with their arms spread, the technician pressed their arms downward as the participant pushed their arms up in the opposite direction.
- Upper trapezius: After the participant was seated with their arms lowered, the technician pressed the shoulder down as the participant pushed up in the opposite direction.
- Erector spinae: While lying on their stomach and keeping their legs fixed, the participant lifted their upper body with maximum force.

After measuring the MVC, the % MVC was calculated using the following formula (Equation (1)) to normalize the muscle activity of the participants:

%MVC = [(RMS_MAX_ − RMS_TASK_)/(RMS_MAX_ − RMS_REST_)] × 100 (1)

#### 2.2.3. Subjective Discomfort

To analyze the subjective discomfort of the participants along with the EMG evaluation, a psychophysical evaluation was conducted simultaneously using Borg’s ratings of perceived exertion scale [17]. The overall subjective discomfort scale consisted of 15 grades (6 = almost no exertion, 7–8 = extremely light, 9–10 = very light, 11–12 = light, 13–14 = somewhat hard, 15–16 = hard, 17–18 = very hard, and 19–20 = extremely hard) and was used to record the immediate response that followed the EMG. Considering the experimental characteristics (i.e., working and break times), 15 grades were analyzed according to the Borg CR10 scale (0 = nothing at all, 0.5 = very very light, 1 = very light, 2 = fairly light, 3 = moderate, 4 = somewhat hard, 5–6 = hard, 7–9 = very hard, 10 = very very hard) [18,19]. In addition, to assess the comfort of the rotational handle type, the discomfort in the hand regions were measured using the locally perceived discomfort (LPD) scale. The hand comprised seven regions, and the scale comprised six grades (0 = no discomfort, 1 = very little discomfort, 2 = moderate discomfort, 3 = high discomfort, 4 = very high discomfort, 5 = extremely high discomfort) [20].

### 2.3. Data Analysis

All the data were subjected to descriptive analyses. After assessing the normality using the Shapiro-Wilk test, the Wilcoxon signed-rank test was used to verify the statistical significance of the EMG results and the subjective discomfort levels with the use of the TB and RHB, which was performed at a significance level of 0.05. The statistical analyses were performed using the SPSS package software (version 27, IBM SPSS Corp., Armonk, NY, USA).

## 3. Results

### 3.1. Comparison of the EMG Results by Basket Types

**Figure 4:**
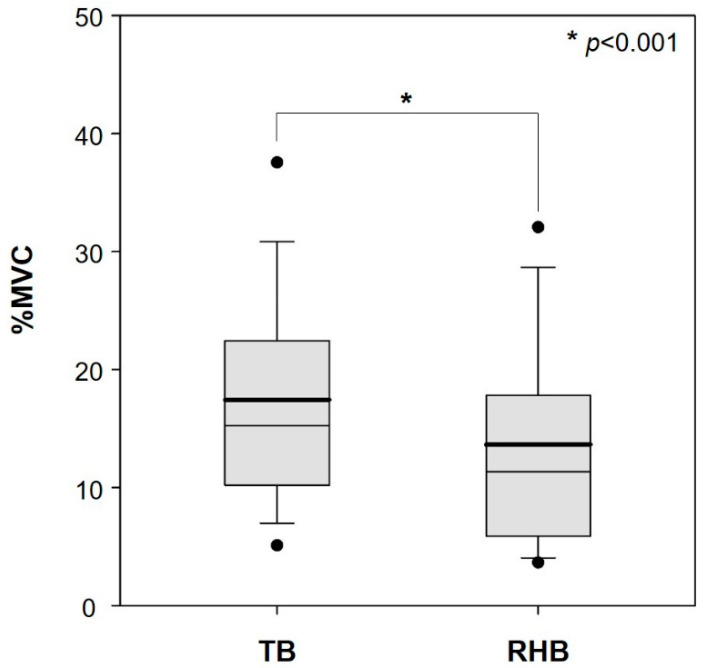
Comparison between the typical harvesting basket (TB) and the harvesting basket with attached the rotational handles (RHB) in terms of total muscle activity (bold line, arithmetic mean; circles, 5th/95th percentiles).

Figure 4 shows the EMG results for all seven muscles. The total muscle activity decreased significantly when the RHB was used (AM, 13.6% MVC; SD, 9.8% MVC) compared with the activity recorded during the use of the TB (AM, 17.4% MVC; SD, 9.7% MVC) (*p* < 0.001).

**Figure 5:**
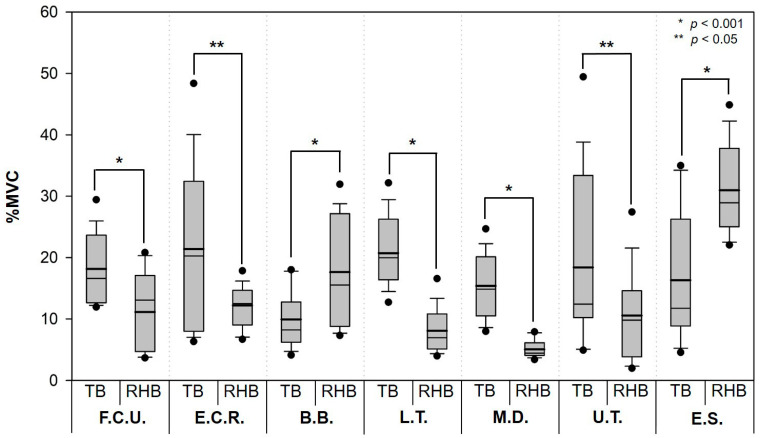
Comparison between the typical harvesting basket (TB) and the harvesting basket with attached the rotational handles (RHB) by seven muscles (bold line, arithmetic mean; circles, 5th/95th percentiles; abbreviations: F.C.U., Flexor Carpi Ulnaris; E.C.R., Extensor Carpi Radialis; B.B., Biceps Brachii; L.T., Lateral Triceps; M.D., Middle Deltoid; U.T., Upper Trapezius; and E.S., Erector Spinae).

Figure 5 shows the evaluation results of the EMG for each muscle area when using the TB and the RHB. The muscle activity of the biceps brachii tended to increase when using the RHB (AM, 17.6% MVC; SD, 9.0% MVC) compared with that noted with the use of the TB (AM, 9.9% MVC; SD, 4.5% MVC), with statistically significant differences in the results (*p* < 0.001). The erector spinae muscle activity also increased with the use of the RHB (AM, 31.0% MVC; SD, 7.1% MVC) compared with that noted with the use of TB (AM, 16.3% MVC; SD, 10.2% MVC). For the remaining five muscle areas, a tendency toward a decrease in % MVC was observed when using the modified basket, with the lateral triceps showing the largest decrease (12.7% MVC) and the flexor carpi ulnaris, the smallest (7.1% MVC), with the decrease being statistically significant compared with that obtained when using the TB (*p* < 0.05). The decrease was also statistically significant for the extensor carpi radialis, middle deltoid, and upper trapezius, whose activities decreased to 9.2% MVC, 12.2% MVC, and 7.9% MVC, respectively (*p* < 0.05).

### 3.2. Results of the Subjective Discomfort Levels by Basket Types

Table 2 shows the results of the subjective discomfort levels according to Borg’s rating of perceived exertion (RPE) and CR10 scales for the four participants after using each basket. The discomfort was reduced when using the RHB compared with the use of the TB (*p* < 0.001). Their response after the use of the TB was that it was somewhat difficult (AM, 13.9 points; somewhat hard); however, the use of the RHB was perceived as being easier (AM, 8.4 points; extremely light).

**Figure 6:**
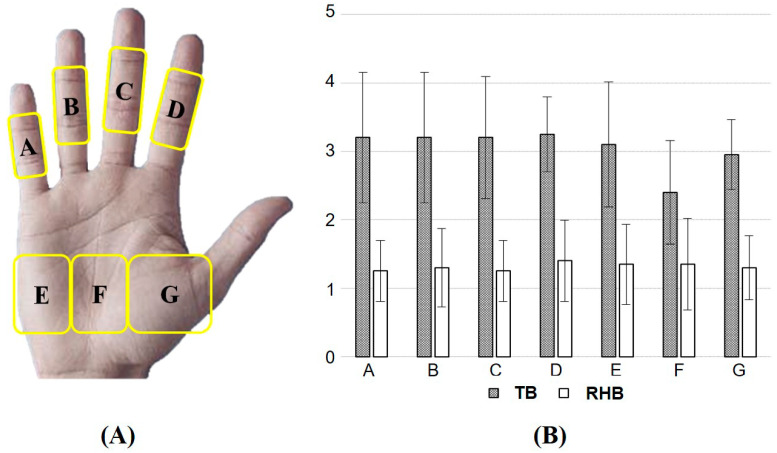
(**A**) Locations (A–G) on the hand, (**B**) Scores of locally perceived discomfort with a typical harvesting basket (TB) and a harvesting basket with attached the rotational handles (RHB).

The LPD scores for each of the seven regions of the hand are shown in Figure 6. The participants’ response to using the TB was that of very high discomfort in all the regions (AM, 3.1 to 3.25 points) except for F and G (AM, 2.4 and 2.95, respectively; moderate discomfort), while they expressed very little discomfort in the entire hand when using the RHB (AM, 1.25 to 1.40), confirming the tendency of a decrease in the score.

## 4. Discussion

In this study, we compared the muscle activity during the lifting of the TB, which is used for various purposes, with that of during the lifting of a basket that was designed ergonomically, with rotatable handles. Furthermore, we also analyzed the subjective discomfort perceived in both cases. The % MVC tended to decrease in most of the seven muscle areas evaluated for muscle activity, except for the biceps brachii and erector spinae. The subjective discomfort levels were evaluated using Borg’s scale for the whole body and an LPD scale for the hands. The participants’ response scores for the two scores were lower when using the RHB compared with those obtained when using the TB.

Harvesting baskets that are used commonly in farmhouses in Korea can weigh more than 30 kg when filled with heavy objects such as agricultural products. When lifting or moving heavy baskets, a worker may lift them high enough for the base of the basket to remain above the pelvis for the convenience of walking. In doing so, however, the wrists cannot be rotated to support the basket or be supported by the shoulder, which can cause a shoulder lift, regardless of the increase in the shoulder angle. If this phenomenon occurs repeatedly, it may lead to disorders such as rotator cuff disease, which may be a high-risk factor for older and female farmers [21,22].

In this study, the use of the RHB showed a tendency to increase the % MVC of the biceps brachii and erector spinae compared with that of the TB. We believe that this increase may have been due to a reduction in any unnecessary force distributed throughout the upper body and an improvement in the unstable posture, leading to an increase in muscle activity. Furthermore, it may also have been due to the influence of the posture or the distance of the heavy object from the body when lifting [23]. As a result, % MVC of the biceps brachii increased in three participants, while the others were similar. On the other hand, % MVC of the erector spinae increased in two participants, while the others were similar. When observing the biceps brachii during the experimental process, while the wrists of the participants with an increased % MVC gripped the rotational handle in the same direction as the upper arm, another participant showed a tendency to become unstable by gripping it with a twist of approximately 5°. In the case of erector spinae, in the two participants with an increased % MVC, the posture of lifting the basket was similar to that of the squat posture; however, the others showed irregular postures. Therefore, it seems that lifting with the right posture affected the erector spinae.

It was confirmed that the overall wrist angle of the participants using the RHB was neutral. As mentioned above, in some cases, the RHB was twisted outward by 5° even when the rotational handle was used. Furthermore, when gripping the handle of the TB, twisting occurred up to a maximum of 40° or more, and when following the criteria of the musculoskeletal system evaluation tool (e.g., rapid upper limb assessment or rapid entire body assessment), it exceeded 15°, and was thus a risk to the wrist [24,25]. Therefore, if workers use a rotational handle, they may reduce the twisting and bending angle of the wrist compared with that noted if they use TBs.

When lifting the TB, the wrists may bend as the basket gets closer to the body due to the shape and position of the handles. Those who used the TB handles tried to support the weight with the upper body because of the load and discomfort on the wrist. Consequently, the working posture may have become unstable, which has been shown to increase other musculoskeletal burdens [26]. However, when using baskets with rotational handles, users did not need to control or maintain a specific distance from the body because the wrist angle is not deflected, and the neutral state could be maintained. We believe that unnecessary force was used to support the upper body with the biceps and erector spinae to increase the muscle activity, since the weight was supported through the arms and the correct posture.

In this experiment, it was verified that muscle activity in the erector spinae tends to increase even without a specific, standardized posture. Studies evaluating the muscle activity of the erector spinae muscle have been conducted both domestically and internationally. The results of a recent study on workers at grocery stores, whose work consisted of lifting and lowering objects, suggested that the % MVC of the erector spinae longissimus increased with the lifting height of weights of approximately 10 kg [27]. In addition, according to a study that evaluated the muscle activity in agricultural work, when it came to manual material handling, the muscle activity of the erector spinae was approximately 50% of the left and right reference voluntary electrical activity [28]. This was approximately 10–15% MVC higher than that when using the RHB that were used in this study; however, it is believed that this was caused by the differences in the weight of the heavy object and the work method.

The use of the RHB is expected to reduce the induction of inadequate working posture and the force exerted on more muscles than necessary. In addition, training will be needed to learn how to lift using the knee to avoid affecting the erector spinae unduly, due to the heavy weight or repetitive work. However, workers handling heavy objects should comply with the appropriate maximum loads. In the US, the lifting maximum load was established as 51 pounds [29]. In Korea, the Ministry of Employment and Labor provided the criteria of musculoskeletal works as follows: lifting objects weighing 25 kg or more 10 times a day; lifting objects weighing 10 kg or more 25 times a day below the knees, above the shoulders or with arms outstretched [30]. In this study, the weight criteria (i.e., approximately 20 kg or more) was not monitored. When handling heavy objects, even with the correct working posture, workers should handle the weight that meets these standards or criteria.

The upper trapezius is a major indicator in the evaluation of neck and shoulder pain related to work [31]. In this study, we observed a tendency toward decreased muscle activity in this muscle area. Therefore, the use of the RHB may be effective in preventing musculoskeletal disorders of the neck and shoulders. It is also expected that this will improve the working postures effectively, avoiding the unnecessary force application to the muscle that occurs when using the TB. The middle deltoid muscle activity decreased significantly, which may have been related to shoulder lifting. Previous studies have reported that the deltoid muscle activity increased as the shoulder angle increased when the arm was raised [32]. Accordingly, the significant decrease in the activity of the middle deltoid when the RHB was used may have been due to a reduction in shoulder lifting.

When the subjective discomfort experienced by the use of the RHB was evaluated using Borg’s RPE scale, it was confirmed that generally it was lower than that when using the TB. This indicated that the use of the RHB is effective in improving unstable postures, such as a shoulder lift and an ulnar deviation. This may have been due to the grip at an inclined angle when using the TB, as the wrist is held at a deviated angle, leading to the weight of the basket becoming concentrated on some of the fingers and the palm. Therefore, we believe that using the RHB may reduce the distribution of pressure applied to certain areas of the fingers and the palm, and ensure even distribution by maintaining the wrists at an angle closer to neutral.

This study aimed to evaluate the EMG and analyze the subjective discomfort levels associated with the use of a new type of harvesting basket with rotational handles. Nevertheless, there were some limitations. This study did not evaluate muscle activity during actual agricultural work, nor did it include a sufficient number of farmers. The effectiveness of the material in the actual field should also be evaluated because of the nature of agricultural work, in which the working method is unclear, and also because one worker may perform several tasks in parallel. Until now, muscle activity has been evaluated for the manual handling of materials such as seedling plates, crops, and agricultural materials; however, there is a need for studies that are focused exclusively on harvesting baskets. Therefore, we believe that this study is meaningful in presenting directions concerning the effect on the musculoskeletal system of the upper body by evaluating the muscle activity when using a basket with rotational handles compared to the use of typical ones. In addition, we confirmed that the wrist angle changed significantly depending on the type of handle on the basket. Therefore, a more precise analysis of the wrist muscles is necessary to evaluate the burden on wrists in the future.

## 5. Conclusions

In this study, muscle activity was evaluated in seven muscle parts of the upper body, and the subjective discomfort levels of the users were investigated, using TBs that were used in various tasks, including agricultural work, and improved baskets with rotational handles. The use of the latter led to an increase in the % MVC in the biceps brachii and erector spinae; however, the % MVC decreased in the remaining five areas. In addition, the users’ responses indicated that the subjective discomfort levels were lower for baskets with rotational handles compared with those noted for the typical ones, and when the LPD score of the hands was investigated in terms of the rotation of the handles, the overall discomfort was found to decrease, regardless of the evaluated hand region. Lifting work can be performed using baskets with rotational handles, which will be helpful in preventing musculoskeletal disorders. In the future, a more accurate evaluation of the effectiveness should be conducted in parallel with actual work and ergonomic and psychophysical analyses targeting a sufficient number of workers.

## Figures and Tables

**Table 1:** Participant information.

Categories	Participants				AM	SD
	F1	F2	M1	M2		
Age (yr)	42	31	33	38	36	5
Weight (kg)	60	54	78	82	68.5	13.6
Height (cm)	162	164.7	185.8	176	172.1	10.9
^(a)^ Length of arm (cm)	104.6	102.5	111.5	108.1	106.7	4
^(b)^ Length of upper arm (cm)	31.6	30.1	35.3	31.5	32.1	2.2
^(c)^ Length of lower arm (cm)	25	25.5	29.5	27.8	27	2.1
^(d)^ Upper arm circumference (cm)	28	27	29.5	33.2	29.4	2.7
^(e)^ Wrist circumference (cm)	15	15	16	16.7	15.7	0.8
^(f)^ Hand length (cm)	18	17.5	19.1	18.7	18.3	0.7
Width of hand (cm)	7.2	7.5	8.5	8.3	7.9	0.6
Thickness of hand (cm)	2.5	2.1	2.8	2.9	2.6	0.4

***Abbreviations*:** AM, Arithmetic Mean; SD, Standard Deviation. ^(a)^ Straight length from lateral shoulder to ulnar styloid through radiale. ^(b)^ Straight length from lateral shoulder to radiale. ^(c)^ Straight length from back olecranon to ulnar styloid. ^(d)^ Circumference of surface on biceps brachii muscle. ^(e)^ Circumference of radial styloid. ^(f)^ Straight length from wrist line to dactylion III.

**Table 2:** Results of the subjective discomfort levels according to the Borg RPE and Borg CR10 scales.

Basket Type	ID	Borg RPE ^(a)^		Estimated Borg CR10 ^(b)^		*p* ^(c)^
		AM	SD	AM	SD	
TB	F1	13.0	0.0	5.5	0.0	<0.001
	F2	15.0	0.0	4.0	0.0	
	M1	13.8	0.4	4.8	0.5	
	M2	13.6	0.5	4.6	0.6	
Sub total		13.9	0.8	4.7	0.3	
RHB	F1	8.2	1.1	1.6	0.6	
	F2	10.2	1.1	2.6	0.6	
	M1	7.0	0.7	0.9	0.6	
	M2	8.0	1.2	1.5	0.6	
Sub total		8.4	1.5	1.7	0.0	

***Abbreviations*:** TB, typical basket; RHB, rotational handle basket; AM, arithmetic mean; and SD, standard deviation. ^(a)^ Borg 6–20 rating of perceived exertion (RPE) scale. ^(b)^ Transferred from Borg RPE score to Borg category-ratio-10 (CR10) scale. ^(c)^ Wilcoxon signed-rank test.

## Data Availability

Not applicable.
